# Rice *NICOTIANAMINE SYNTHASE 2* expression improves dietary iron and zinc levels in wheat

**DOI:** 10.1007/s00122-016-2808-x

**Published:** 2016-10-08

**Authors:** Simrat Pal Singh, Beat Keller, Wilhelm Gruissem, Navreet K. Bhullar

**Affiliations:** 1 Plant Biotechnology, Department of Biology, ETH Zurich, Zurich, Switzerland; 2 Institute of Plant Biology, University of Zurich, Zurich, Switzerland

## Abstract

**Key message:**

**Iron and zinc deficiencies negatively impact human health worldwide. We developed wheat lines that meet or exceed recommended dietary target levels for iron and zinc in the grains. These lines represent useful germplasm for breeding new wheat varieties that can reduce iron and zinc deficiency**-**associated health burdens in the affected populations.**

**Abstract:**

Micronutrient deficiencies, including iron and zinc deficiencies, have negative impacts on human health globally. Iron-deficiency; anemia affects nearly two billion people worldwide and is the cause of reduced cognitive development, fatigue and overall low productivity. Similarly, zinc deficiency causes stunted growth, decreased immunity and increased risk of respiratory infections. Biofortification of staple crops is a sustainable and effective approach to reduce the burden of health problems associated with micronutrient deficiencies. Here, we developed wheat lines expressing rice *NICOTIANAMINE SYNTHASE 2* (*OsNAS2*) and bean *FERRITIN* (*PvFERRITIN*) as single genes as well as in combination. *NAS* catalyzes the biosynthesis of nicotianamine (NA), which is a precursor of the iron chelator deoxymugeneic acid (DMA) required for long distance iron translocation. FERRITIN is important for iron storage in plants because it can store up to 4500 iron ions. We obtained significant increases of iron and zinc content in wheat grains of plants expressing either *OsNAS2* or *PvFERRTIN*, or both genes. In particular, wheat lines expressing *OsNAS2* greatly surpass the HarvestPlus recommended target level of 30 % dietary estimated average requirement (EAR) for iron, and 40 % of EAR for zinc, with lines containing 93.1 µg/g of iron and 140.6 µg/g of zinc in the grains. These wheat lines with dietary significant levels of iron and zinc represent useful germplasm for breeding new wheat varieties that can reduce micronutrient deficiencies in affected populations.

**Electronic supplementary material:**

The online version of this article (doi:10.1007/s00122-016-2808-x) contains supplementary material, which is available to authorized users.

## Introduction

Micronutrients are essential in the human diet because they are required for key metabolic reactions and biological functions. A large fraction of the global human population suffers from micronutrient deficiencies, which has a negative impact on well-being and economic development (Muthayya et al. 2013). The most common deficiencies include iron, zinc, iodine, vitamin A, vitamin B-12, riboflavin, vitamin D, and vitamin E deficiencies (Allen et al. 2009). Iron and zinc deficiencies are among the most widespread micronutrient deficiencies. Iron deficiency is the leading cause of iron-deficiency anemia (IDA), which is prevalent world wide in both the developed and developing countries (WHO 2016). IDA affects cognitive development, decreases immune function, and causes higher mortality of mothers and children at birth. Children as well as pregnant and non-pregnant women are at a higher risk with approximately 43, 38 and 29 % of the population, respectively, affected with IDA (Stevens et al. 2013). Zinc deficiency is associated with diseases such as diarrhea, pneumonia and malaria (WHO 2002). The main clinical symptoms include growth retardation, cell-mediated immune dysfunction and cognitive impairment (Prasad 2014). Iron and zinc deficiencies are often associated with low micronutrient content in staple foods, poor micronutrient absorption from food, and dependence of the affected populations on simple and monotonous diets.

The three recommended approaches to overcome micronutrient deficiencies include supplementation, food fortification and biofortification. Micronutrient supplementation, which has been implemented in different parts of the world, is not always successful. For example, a nutritional anemia control program was implemented in India in 1970 but had only limited impact mainly because of logistical problems, poor compliance, and lack of sufficient funds (Mayer et al. 2008). This suggests that supplementation is not a sustainable preventive solution, but rather a short-term curative measure. Food fortification with iron is difficult because iron compounds such as FeSO_4_ cause food to have a generally unacceptable flavor and color, thus making it unpalatable (Abbaspour et al. 2014; Hurrell 2002). Biofortification of staple crops, i.e., enhancing the nutritional content of the edible parts, offers the most sustainable method to overcome micronutrient deficiencies.

Wheat (*Triticum aestivum*) is grown on 219 million hectares (*mha*) with a production of over 715 metric tonnes (MT) and is one of the most widely grown and consumed cereals globally (FAOSTAT 2013). Several billion people around the world depend on wheat for protein, starch, fiber, and various essential micronutrients (Shewry 2009). However, most of the bread wheat varieties do not contain sufficient iron and zinc in the grains to meet the daily dietary recommendations for these nutrients (Zhao et al. 2009). Therefore, biofortification of wheat for iron and zinc can greatly benefit global human health. According to the HarvestPlus program initiated by the Consultative Group for International Agricultural Research (CGIAR), 59 μg/g (dry weight) of iron and 38 μg/g (dry weight) of zinc is required in the wheat grains to meet 30 and 40 % of the estimated average requirement (EAR) of an adult diet, respectively (Bouis et al. 2011). To date, conventional breeding has achieved little or no success in enhancing the iron content of wheat. Screening of wheat germplasm showed a negative correlation between grain yield and micronutrient content (Amiri et al. 2015; Zhao et al. 2009), which might be the consequence of plant breeding focusing mainly on agronomic yield than nutritional quality in the past. Therefore, increasing grain iron and zinc content in wheat while maintaining yield has been difficult to achieve via conventional breeding. Biofortification utilizing gene technology offers a multi-dimensional approach to enrich the required micronutrients specifically in the grains without altering overall yield (Bhullar and Gruissem 2013).

Iron is important for both plants and animals. However, excessive iron accumulation is toxic to cells, and iron homeostasis is tightly regulated in animals and plants. Thus, a careful choice of strategies is required to increase iron content in cereal grains (Grusak et al. 1999). Cereals, including rice and wheat, use a chelation-based strategy (also known as Strategy II) for iron uptake (Marschner and Romheld 1994). Mugineic acid family phytosiderophores (PS) are released into the soil where they chelate the ferric form of iron, forming a PS-Fe^3+^ complex, which is subsequently transported into the roots by specific transporters. The PS are synthesized from S-adenosyl-l-methionine via a conserved pathway of reactions catalyzed by nicotianamine synthase (NAS), nicotianamine aminotransferase (NAAT), and deoxymugineic acid synthase (DMAS) (Kobayashi and Nishizawa 2012). Based on this knowledge various single gene and multigene strategies have been developed in rice to improve iron content in the endosperm (Bhullar and Gruissem 2013). Masuda et al. (2009) showed that constitutive overexpression of the *HvNAS1* gene in rice resulted in a threefold increase of iron in the rice endosperm. Similarly, constitutive overexpression of *OsNAS2* increased iron content in polished rice by 2- to fourfold (Johnson et al. 2011; Lee et al. 2012). In addition to iron, the zinc content was also increased in polished grains of *NAS* overexpressing lines (Johnson et al. 2011; Wirth et al. 2009). The Nicotianamine (NA) and PS can also bind Zn, in addition to Fe, thus explaining the accompanied increases in zinc content in the lines overexpressing *NAS* (Schaaf et al. 2004). Transformation of cereals with *FERRITIN* to increase the iron content has also been reported (Borg et al. 2012; Vasconcelos et al. 2003). FERRITIN is a complex of 24 protein subunits arranged to form a hollow structure that can store up to 4500 ferric molecules (Harrison and Arosio 1996). In rice, *FERRITIN* expressed under the control of an endosperm-specific promoter increased iron content in polished rice grains by 2- to 3.7-fold (Goto et al. 1999; Lucca et al. 2001; Oliva et al. 2014; Qu et al. 2005; Vasconcelos et al. 2003). Moreover, constitutive expression of soybean *FERRITIN* (*GmFERRITIN*) increased iron content in the leaves of wheat (Drakakaki et al. 2000). Borg et al. (2012) overexpressed endogenous wheat *FERRITIN (TaFERRITIN1*-*A)* under the control of the native endosperm-specific promoter of *TaFERRITIN1*-*A*, and reported an increase of 50–80 % of iron content in wheat grains. In rice, expression of both *FERRITIN* and *NAS* synergistically increases the grain iron content further. Endosperm-specific expression of bean *PvFERRITIN* and constitutive expression of Arabidopsis *AtNAS1* from a single construct increased iron content in rice endosperm by sixfold (Wirth et al. 2009).

To achieve the recommended level of iron in wheat grains for healthy human diets (i.e., minimum 59 µg/g iron/dry weight; Bouis et al. 2011) it is necessary to increase iron content by 100 % in the major wheat cultivars. We have engineered bread wheat with a bean *FERRITIN* (*PvFERRITIN*) gene expressed under the control of the endosperm-specific rice *GLOBULIN* (*OsGLOBULIN*) promoter and the rice *NAS2* (*OsNAS2*) gene expressed under the control of constitutive-promoter maize *UBIQUITIN* (*ZmUBIQUITIN*) as single gene constructs and as a combination of both genes. Expression of *PvFERRITIN* or *OsNAS2* alone as well as in combination significantly increased iron and zinc levels in wheat grains, with several lines surpassing the recommended target levels for both iron and zinc content.

## Materials and methods

### Transformation vectors

**Fig. 1:**
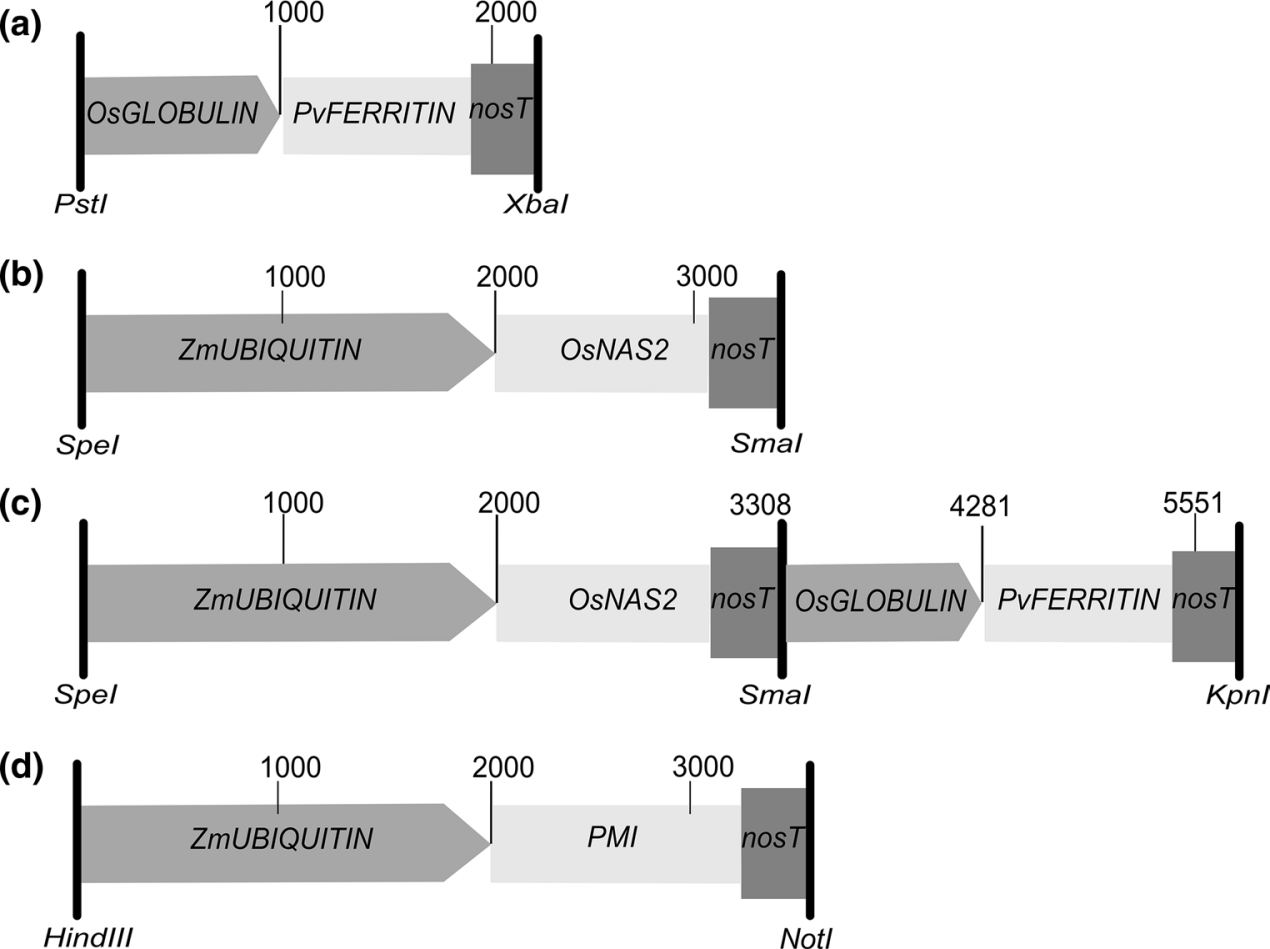
Schematic illustration of the DNA constructs used for transformation. **a** *PvFERRITIN* (transformed in Fer-BW *lines*). **b** *OsNAS2* (transformed in Nas-BW *lines*). **C** *PvFERRITIN*-*OsNAS2* (transformed in FerNas-BW *lines*). **D** ZmUBIQUITIN-*PMI* (co-transformed with constructs **a**, **b** and **c**). *Numbers* indicate length of the gene cassette in base pairs. For details see “Materials and methods”

*PvFERRITIN* (X58274) under the control of the rice endosperm-specific *OsGLOBULIN* promoter (Wirth et al. 2009) was excised from the parent plasmid using *Xba1* and *Pst1* and was used for transformation of wheat. For generation of the construct combining both *PvFERRITIN* and *OsNAS2,* we incorporated *KpnI* restriction site upstream of the *OsGLOBULIN* promoter and *SmaI* restriction site at 3′ of *nopaline synthase* (*NOS*) gene terminator (*nosT*) in the *PvFERRITIN* construct, and cloned it to the Pjet1.2 vector, generating *PvFERRITIN*-Pjet1.2 plasmid. The rice *OsNAS2* (LOC_Os03g19420.2) gene was commercially synthesized along with *nosT* from GenScript^®^ (http://www.genscript.com). *SpeI*, *HindIII*, and *BamHI* restriction sites were inserted upstream of *OsNAS2*, and *SmaI* was inserted at 3’end, and cloned into the *puc57* vector, generating the *OsNAS2*-*puc57* construct. The *ZmUBIQUITIN* promoter from *pAHC17* (Christensen and Quail 1996) was excised using *HindIII* and *BamHI*, and was inserted into the *OsNAS2*-*puc57* construct. The entire *OsNAS2* cassette was then excised using *SpeI* and *SmaI* restriction sites, and was inserted to the *PBSKII*(−) vector, generating *OsNAS2*-*PbskII*(−). Similarly, the *PvFERRITIN* cassette was excised from the *PvFERRITIN*-Pjet1.2 construct using *KpnI* and *SmaI*, and cloned into the *OsNAS2*-*PbskII*(−) construct, finally generating *PvFERRITIN*-*OsNAS2*. For transformation, vector backbone-free gene cassettes were co-transformed along with the *PHOSPHOMANNOSE ISOMERASE* (*PMI*) selectable marker gene expressed under the control of the *ZmUBIQUITIN* promoter (Fig. 1) (Brunner et al. 2011).

### Wheat transformation, plant growth and characterization

Hexaploid spring wheat cultivar Bobwhite SH 98 26 was transformed using particle gun bombardment (Brunner et al. 2011; Pellegrineschi et al. 2002). Overall, approximately 6500 immature embryos were co-transformed with *PMI* and *PvFERRITIN*, *PMI* and *OsNAS2*, or *PMI* and *PvFERRITIN*-*OsNAS2* generating transformed lines named *PvFERRITIN*-Bobwhite (hereafter Fer-BW), *OsNAS2*-Bobwhite (hereafter Nas-BW), and *PvFERRITIN*-*OsNAS2*-Bobwhite (hereafter FerNas-BW), respectively. Selection of the transformed plants was performed on culture media containing mannose during the regeneration phase (Wright et al. 2001). Plants were later grown in commercial soil (Klasmann-Deilmann GmbH, Germany) in the greenhouse in 16 h light/22 °C and 8 h dark/18 °C, and a humidity of 60 %. Genomic DNA was isolated from 3-week-old seedlings in the T0-, T1-, T2- and T3-generation (Stein et al. 2001; Vasudevan et al. 2014). PCR was used to confirm the presence of the transgenes using gene specific primers (Table. S1). PCR-positive lines were further selected based on the iron content in the subsequent generations. Southern blot analysis was performed using a P^32^-labelled probe to select lines with a single copy insert (Fig. S1) in T0-generation plants (Green and Sambrook 2012). The probes for *PvFERRITIN*, *OsNAS2*, and *PMI* were generated using gene specific primers (Table. S1). For phenotypic characterization of transformed wheat lines, parameters including days to flowering (DTF), plant height, 1000 grain weight (1000 GW), and tiller number were recorded.

### Metal ion measurements

Plants were harvested 6 weeks after flowering and spikes were dried at 37 °C for 3 days. Grain samples were de-husked and grounded for metal ion measurements. Additionally, ground grain samples were fractioned using a 250 μm nylon sieve to obtain sieved flour (Borg et al. 2012), referred to as ‘flour’ hereafter. Two hundred mg of sample was boiled in 15 ml of 65 % v/v HNO_3_ solution at 120 °C for 90 min. Three ml of 30 % v/v H_2_O_2_ was added and boiled at 120 °C for 90 min. Metal concentrations were determined using inductively coupled plasma-optical emission spectroscopy (ICP-OES) (Varian Vista-MPX CCD Simultaneous ICP-OES). The wavelength used for iron, zinc, copper, manganese, and magnesium was 238.204, 213.857, 324.754, 257.610, and 285.213, respectively. The iron concentrations were recorded in T2, T3 and T4 grains, and the seeds from plants with highest iron concentration were used to grow the next generation of plants (Fig. S2). Data were analyzed using the Student’s *t* test to determine statistically significant differences among the transformed lines and their respective controls.

### Quantitative real-time PCR

Quantitative real-time PCR (qRT-PCR) was performed to assess the expression of the transgenes in the transgenic lines. Total RNA was extracted from leaves and grains collected 18 days after flowering (DAF) in the T3 generation plants. Leaf RNA was extracted using Trizol^®^ reagent (Invitrogen, USA), and the RNA was treated with DNase I (Thermo Fisher Scientific, Inc., USA) to remove genomic DNA contamination. cDNA synthesis was done using the RevertAid™ first strand cDNA synthesis kit (Thermo Fisher Scientific, Inc., USA). qRT-PCR was performed on the 7500 FAST Real Time PCR system (Applied Biosystems, Inc., USA). The total reaction volume of 20 µl included 1 µl cDNA, 0.4 µl forward primer, 0.4 µl reverse primer, 10 µl Sybrgreen Mastermix (Applied Biosystems, Ltd., USA), and 8.2 µl H_2_O. Primers were designed using a CLC Genomics Workbench (Table S1). For data normalization, *Ta.22845* was used as reference gene that encodes for ATP-dependent 26S proteasome regulatory subunit (Paolacci et al. 2009), and it expresses both in the flag leaves as well as the grains of wheat (own unpublished data). The *C*t value was obtained from 7500 Fast System Software. The data normalization was done as described by Liu et al. (2011).

## Results

### Seed specific expression of *PvFERRITIN* and constitutive expression of *OsNAS2* in wheat

**Fig. 2:**
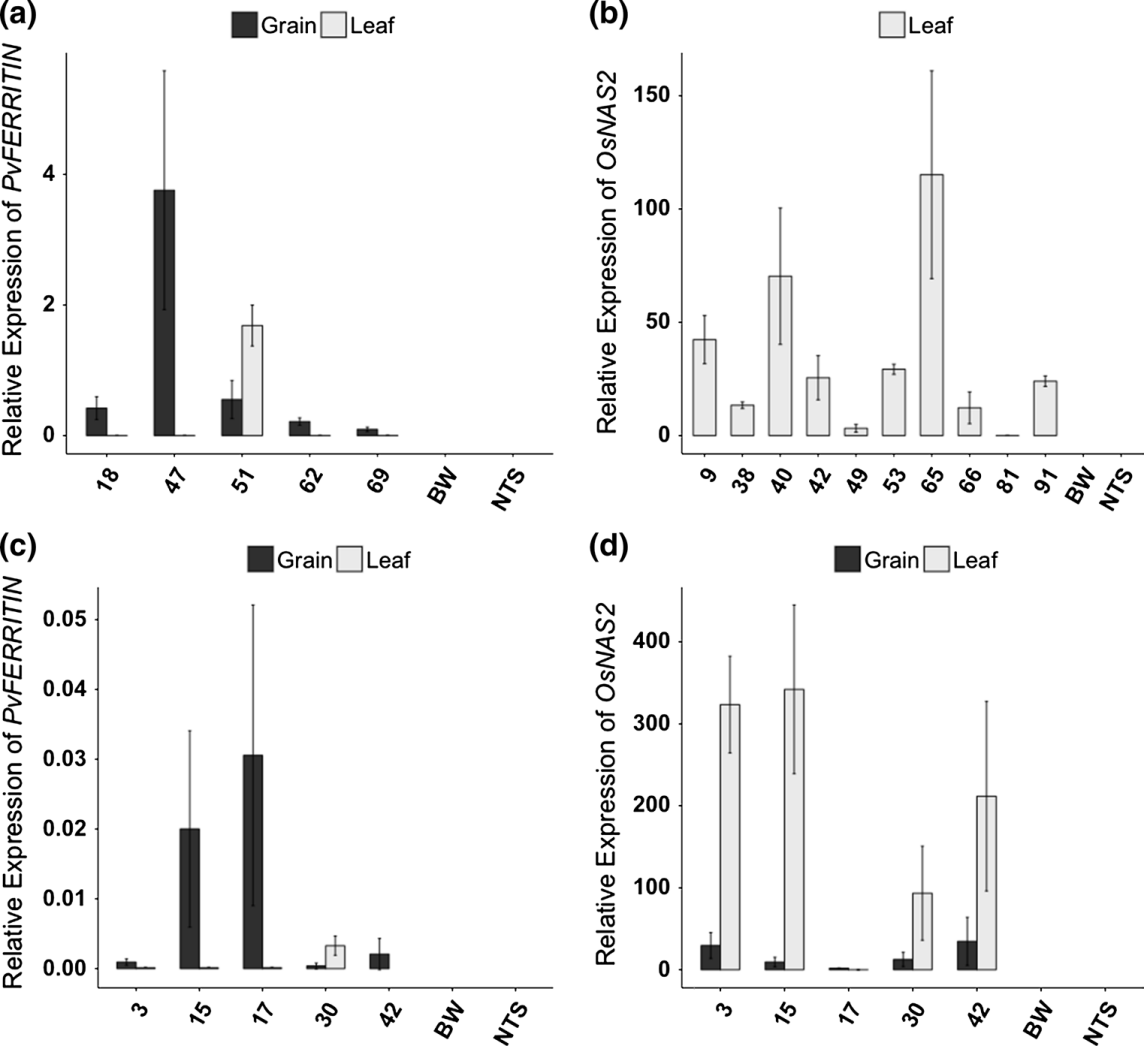
Relative expression of transgenes *PvFERRITIN* and *OsNAS2* at 18 days after flowering (DAF) in T3-generations. **a** Relative expression of *PvFERRITIN* in grains and leaves of Fer-BW *lines*. **b** Relative expression of *OsNAS2* in leaves of Nas-BW *lines*. **c** Relative expression of *PvFERRITIN* in FerNas-BW *lines*. **d** Relative expression of *OsNAS2* in FerNas-BW *lines*. No expression of *PvFERRITIN* and *OsNAS2* was observed in Bobwhite (BW) and non-transgenic sibling (NTS) controls. The data were normalized to the endogenous expression of *Ta.22845*. Values are the average of three biological replicates (±SD)

Single insertion Fer-BW, Nas-BW, and FerNas-BW transgenic lines (Figs. 1, S1) were analyzed for transgene expression in grains and leaves. *PvFERRITIN* was specifically expressed in the grains of all Fer-BW and FerNas-BW lines, except for two lines, Fer-BW 51 and FerNas-BW 30, which also showed expression in leaves (Figs. 2a, c, S3). These results confirm the seed-specific expression of *PvFERRITIN* under the control of the rice *OsGLOBULIN* promoter in most wheat lines. *OsNAS2*-specific primers (Table S1) were used to analyze the expression of the transgene in Nas-BW leaves and both leaves and grains of FerNas-BW lines (Fig. 2b, d). All Nas-BW lines showed *OsNAS2* expression in the leaves. In FerNas-BW lines *OsNAS2* expression was also detected in grains, except for FerNas-BW 17. However, *OsNAS2* expression was many folds higher in leaves than grains, indicating that the *ZmUBIQUITIN* promoter is not very active in wheat endosperm.

### Expression of *PvFERRITIN* increases iron and zinc content in whole grains and flour

**Fig. 3:**
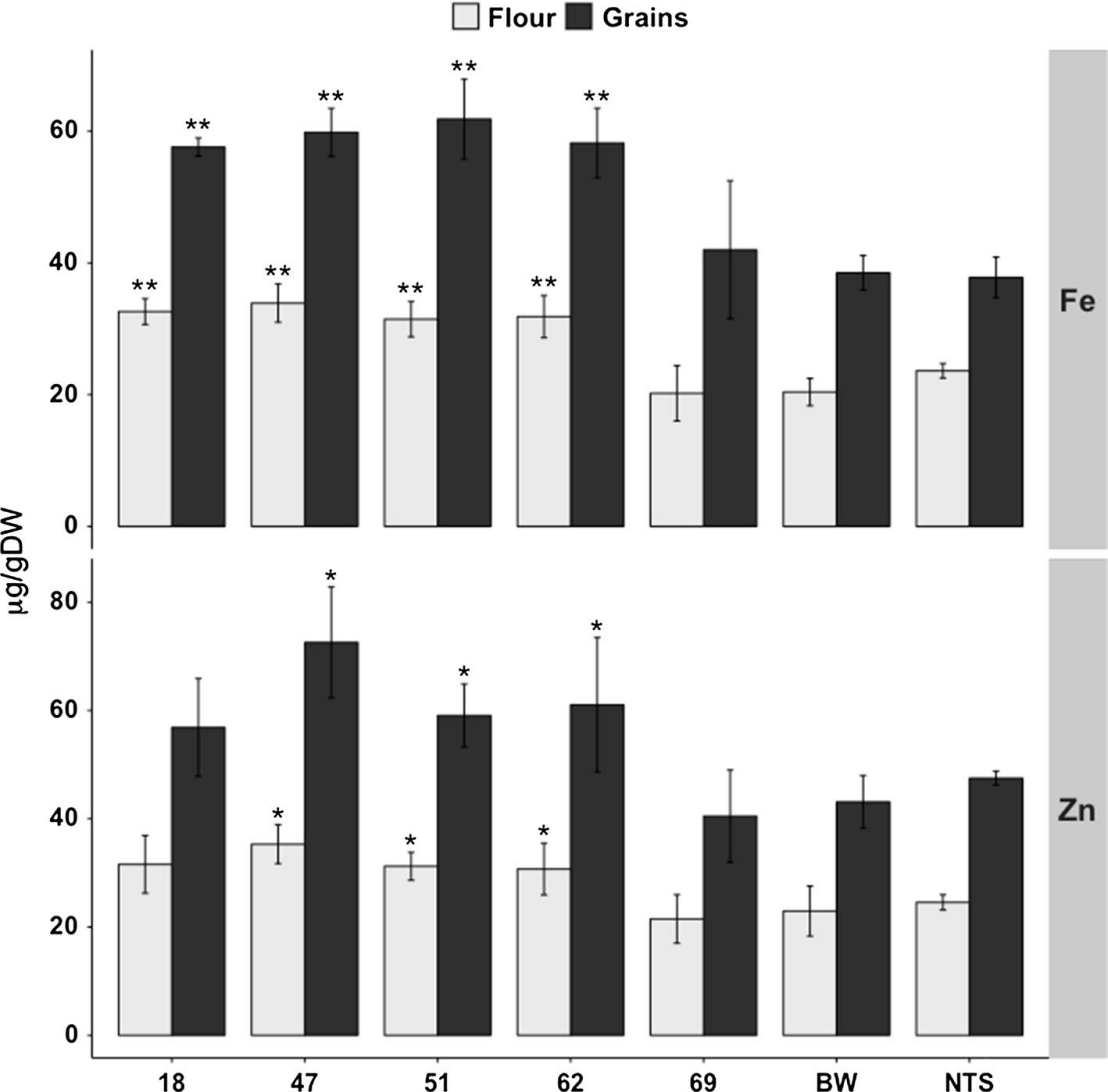
Iron (Fe) and zinc (Zn) content in T4 grains and flour of lines expressing *PvFERRITIN* (Fer-BW). Values are the mean of three biological replicates (±SD). *Black asterisks* above the *bars* indicate statistically significant values calculated using Student’s *T* test, in comparison to the control line Bobwhite (BW) (*P < 0.05, **P < 0.01).* NTS* non-transgenic sibling

The single insertion Fer-BW lines 18, 47, 51, 62, and 69 were analyzed for their metal content. Iron content was significantly increased in all lines except Fer-BW 69. Iron content in the T4 grains of these lines ranged between 42.0 and 61.9 µg/g DW, as compared to 38.5 µg/g DW in Bobwhite (Fig. 3). Fer-BW 51 had the highest iron content (61.9 µg/g) with an increase of 1.6-fold as compared to the control grains. The iron content in the flour (the endosperm of the grains) of Fer-BW transgenic lines ranged from 20.2 to 33.9 µg/g DW compared to 20.4 µg/g DW in Bobwhite. Flour from grains of line 47 had the highest iron content, with a 1.7-fold increase (33.6 µg/g) as compared to the control (Fig. 3). In addition, whole grains of lines 18, 47, 51 and 62 had significantly increased zinc content ranging from 59.1 to 72.6 µg/g DW as compared to 43.1 µg/g DW in Bobwhite (Figs. 3, S4). Zinc content in the flour ranged from 30.7 to 35.3 µg/g DW in the transformed lines as compared to 23.0 µg/g DW in the control. Line Fer-BW 47, which contained the highest iron content in the flour, also contained the highest zinc content in whole grains (72.6 µg/g) as well as in flour (35.3 µg/g), representing 1.7-fold and 1.5-fold increases, respectively, as compared to the control. In addition, copper, manganese, and magnesium levels were also measured in whole grains and flour. Copper was increased in grains of four of the Fer-BW lines, except for line 69 (Fig. S5), and ranged from 6.9 to 8.3 µg/g DW compared to 6.5 µg/g DW in the control line. A similar trend was observed in the copper content of flour which ranged from 4.8 to 5.7 µg/g DW in the transformed lines, compared to 3.91 µg/g DW in Bobwhite. With few exceptions, manganese and magnesium content was not altered in the transformed lines (Fig. S5). Phenotypic greenhouse performance of the Fer-BW lines were similar to Bobwhite for plant height and 1000 GW, while they showed some variation for days to flowering (DTF) and tiller number (Table. S2).

### *OsNAS2* overexpression increases iron and zinc content to dietary significant levels

**Fig. 4:**
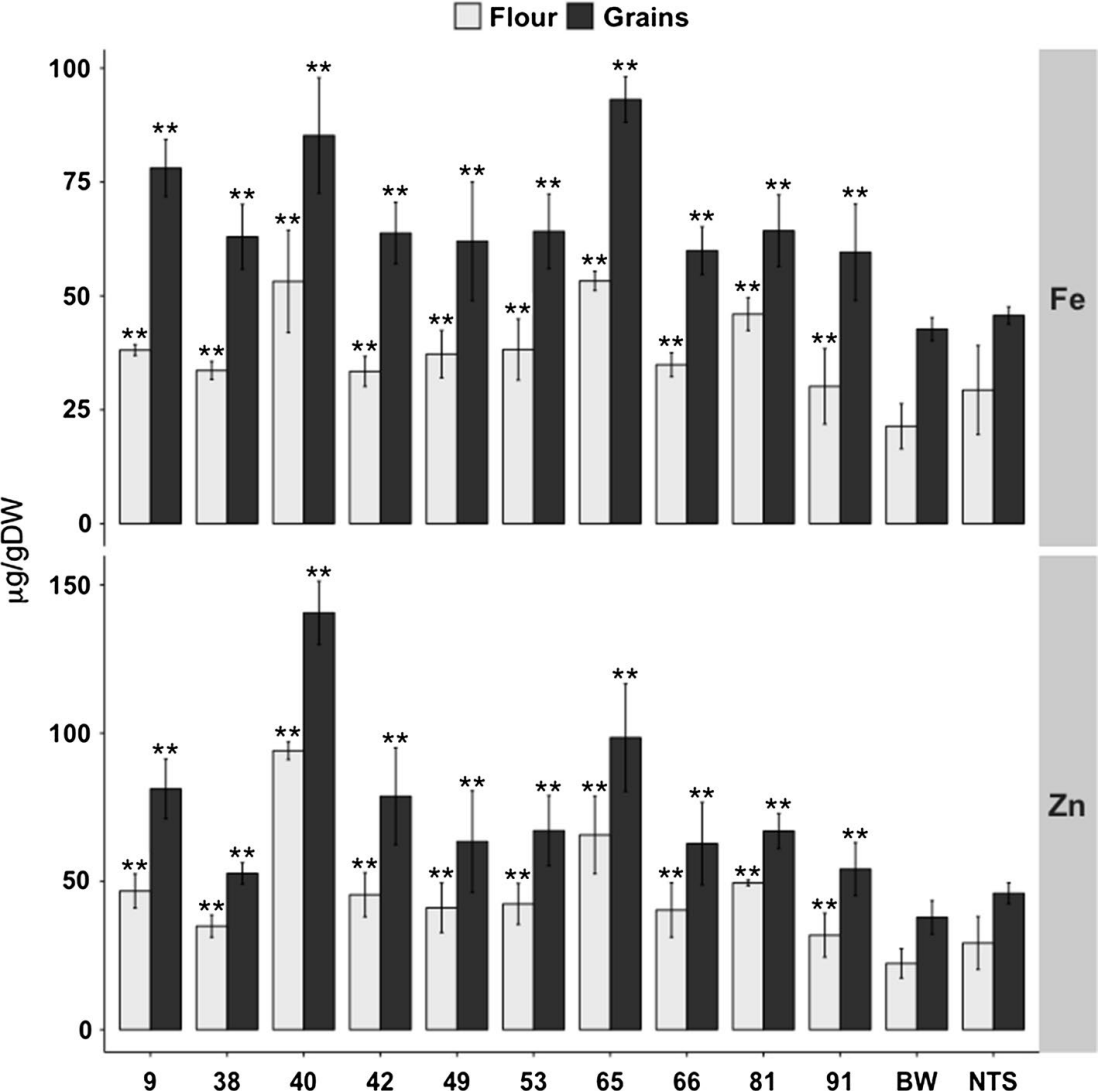
Iron (Fe) and zinc (Zn) content in T4 grains and flour of lines expressing *OsNAS2* (Nas-BW). Values are the mean of three biological replicates (± SD). *Black asterisks* above the *bars* indicate statistically significant values calculated using Student’s *T* test, in comparison to the control line Bobwhite (BW) (*P < 0.05; **P < 0.01). *NTS* non-transgenic sibling

Ten single insertion Nas-BW lines expressing rice *OsNAS2* had significant increases in iron and zinc content in comparison to Bobwhite (Fig. 4). The iron content in T4 whole grains ranged from 59.6 to 93.1 µg/g DW, as compared to 42.7 µg/g DW in the control. Moreover, iron content in the flour of these lines ranged from 30.1 to 53.3 µg/g DW, as compared to 21.4 µg/g DW in the control. Line 65 had the greatest increase in iron (2.1-fold) in whole grains and a 2.5-fold increase in the flour as compared to Bobwhite. The Nas-BW lines had relatively higher iron content in comparison to Fer-BW lines and all of these lines also surpass the suggested target levels of 59 µg/g DW iron in whole grains to meet the 30 % estimated average requirement of human diets. The iron increases in the NAS-BW lines correlated well with the transgene expression (Fig. 2) in these plants, with line 65 having the highest *OsNAS2* expression in leaves.

The zinc content in whole grains of the transformed lines ranged from 54.2 to 140.6 µg/g DW and in the flour from 31.9 to 94.1 µg/g DW, as compared to 37.9 and 22.4 µg/g DW in Bobwhite, respectively (Fig. 4). Line 40 had the greatest zinc levels with a 3.7-fold increase (140.6 µg/g) in whole grains and a 4.2-fold increase (94.1 µg/g) in flour, as compared to control. Increases in copper, magnesium, and manganese content were also measured in most of the transformed lines (Fig. S6). All lines had significantly higher copper content in whole grains as well as flour. The manganese content also increased significantly in whole grains and flour in most of the lines except in whole grains of lines 38, 53, 66 and 81 and flour of line 66. As an exception, line 91 showed significantly lower manganese content in both whole grains and flour. The magnesium content in whole grains as well as flour was significant increased except in whole grains of lines 42 and 66 and flour of line 91.

Importantly, most of the Nas-BW lines were phenotypically indistinguishable from Bobwhite for DTF, plant height, 1000 GW, and tiller number (Table 1). Lines 40 and 91 flowered earlier, and 1000 GW of lines 40 and 42 was significantly increased but decreased in line 81 as compared with Bobwhite.

**Table 1:** Phenotypic performance of greenhouse-grown T3 generation Nas-BW transgenic lines

Plant line	Days to flowering	Height (cm)	1000 GW (g)	Tiller
9	99.3 ± 2.9	53.7 ± 4	30.1 ± 3	15.7 ± 1.5
38	105.3 ± 6.4	58.5 ± 4.9	23.3 ± 3.2	17 ± 1.7
40	92 ± 3.5**	36.3 ± 2.8	34.6 ± 1.7*	20.7 ± 3.1
42	97.7 ± 3.5	49.1 ± 1.6	37.8 ± 1.8**	20.7 ± 6.1
49	107.3 ± 5	54.8 ± 1.5	29.6 ± 3.7	20.3 ± 1.2
53	106.3 ± 4.7	58.9 ± 10.3	24.8 ± 4.1	18.7 ± 3.5
65	98.7 ± 5.7	58.5 ± 1.7	27.7 ± 6.7	16 ± 2.6
66	97.3 ± 12.7	55.8 ± 5.3	29 ± 5.9	17 ± 3.5
81	100.7 ± 4.5	52.8 ± 4.4	18.8 ± 3**	16 ± 2.6
91	89 ± 4.4**	47.2 ± 2.3	29.9 ± 3.9	15.3 ± 4
NTS	109.7 ± 7.8	50.3 ± 2.7	31.5 ± 0.5	14.7 ± 5.5
BW	101.7 ± 0.5	49.6 ± 1.9	29.2 ± 1.9	16 ± 3

Values are the average of three biological replicates (±SD). Transgenic plants were compared to Bobwhite (BW). Black and red asterisks indicate statistically higher and lower significant values calculated using Student’s *T* test, respectively (*P < 0.05, **P < 0.01)

### Iron and zinc levels in lines expressing both *PvFERRITIN* and *OsNAS2*

**Fig. 5:**
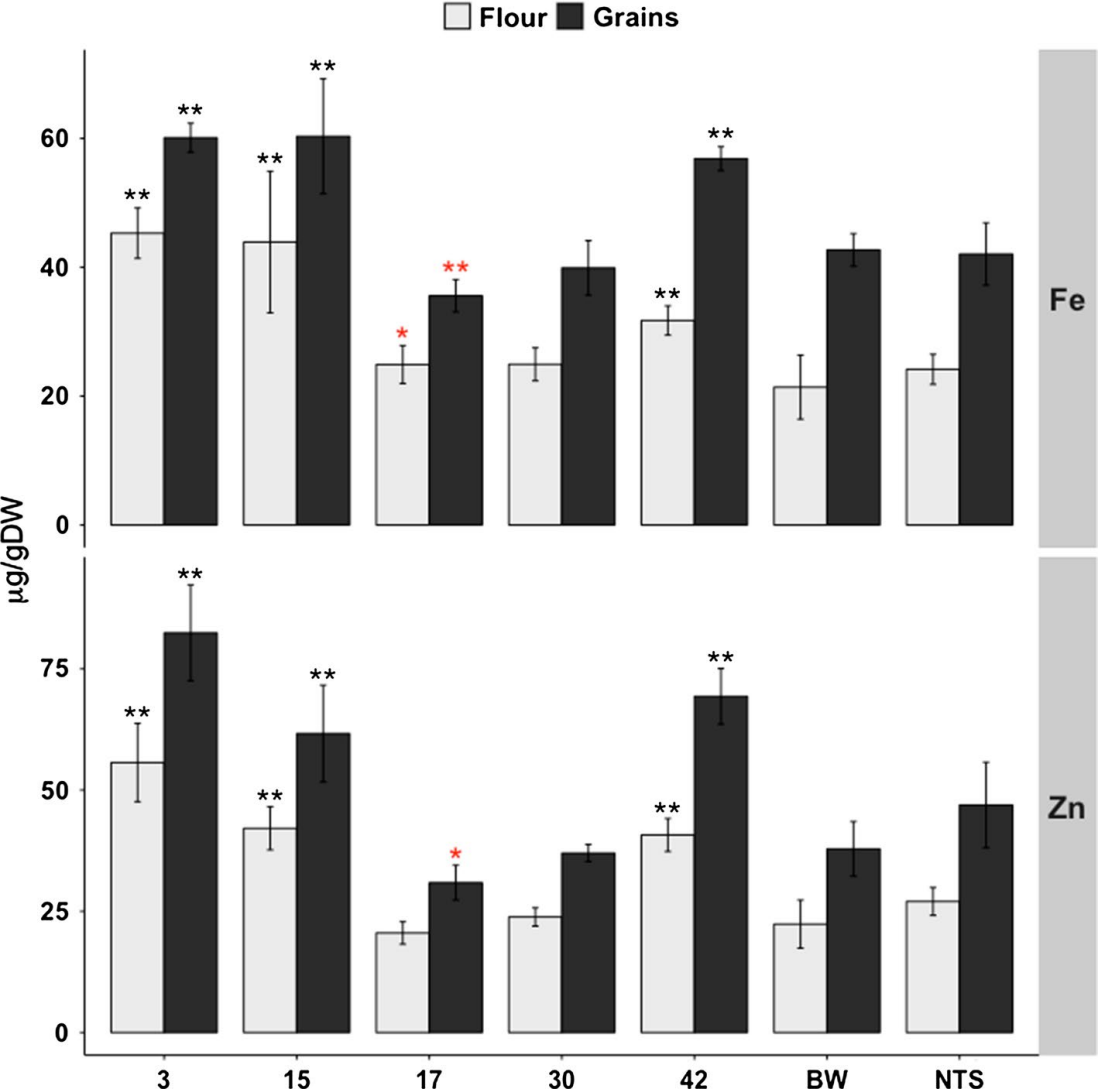
Iron (Fe) and zinc (Zn) content in T4 grains and flour of lines expressing *PvFERRITIN*-*OsNAS2* (FerNas-BW). Values are the mean of three biological replicates (±SD). *Black* and *red asterisks* above the *bars* indicate statistically higher and lower significant values calculated using Student’s *T* test, respectively, in comparison to the control line Bobwhite (BW) (*P < 0.05; **P < 0.01). *NTS* non-transgenic sibling

Wheat lines expressing *PvFERRITIN* and *OsNAS2* from a single construct (FerNas-BW lines) were analyzed to determine if the transgenes act synergistically leading to higher iron and zinc increases in comparison to plants expressing single genes (Fer-BW and Nas-BW lines). The iron content in whole grains of five single insertion FerNas-BW lines ranged from 35.6 to 60.3 µg/g DW, as compared to 42.7 µg/g DW in Bobwhite (Fig. 5). The flour of these transgenic lines had iron content in the range of 24.9–45.3 µg/g DW, compared to 21.4 µg/g DW in Bobwhite. FerNas-BW lines had a maximum 1.7-fold increased of iron (60.3 µg/g in line 15) in whole grains and of 1.8-fold increased iron in the flour (45.3 µg/g in line 3), as compared to Bobwhite. Among the FerNas-BW lines, line 3 had the highest zinc content in whole grains (82.4 µg/g DW) as well as in flour (55.7 µg/g DW) (Fig. 5). However, in contrary to the expectations, these increases for iron and zinc content are smaller than those observed in plants expressing either *PvFERRITIN* or *OsNAS2* alone. These lower increases in iron and zinc content possibly correlate to a relatively lower level of *FERRITIN* expression in the FerNas-BW lines as compared to that in the Fer-BW lines.

Copper, manganese and magnesium contents were variable among the different FerNas-BW lines (Fig. S7). Lines 3, 15, and 42 showed significantly increased copper and magnesium content in both whole grains as well as the flour. In contrast, lines 17 and 30 had reduced copper content in comparison to the control. Manganese content was also significantly higher in lines 3 and 15 for both the whole grains and the flour, as compared with Bobwhite. FerNas-BW lines were phenotypically similar to Bobwhite for DTF, 1000 GW and tiller number as well as plant height, except for line 42 which was significantly taller (Table S3).

## Discussion

The development of high yielding farmer-preferred wheat cultivars with increased micronutrient content in the grains can improve the well-being of millions of malnourished humans around the globe. While increases for some micronutrients could be achieved via conventional breeding, the improvements for many other micronutrients including iron, vitamin A and folate are difficult to achieve (Bhullar and Gruissem 2013; Mayer et al. 2008; Slamet-Loedin et al. 2015). In 2015, zinc biofortified wheat varieties were released in India and Pakistan (HarvestPlus 2015a, b). Zinc fertilizer application has also been suggested as a useful strategy for zinc biofortification of wheat (Cakmak 2008). Similar agronomic practices as well as conventional breeding approaches, however, have not been successful in achieving the recommended increase of iron content in wheat grains. Furthermore, it is difficult to implement targeted fertilizer application strategies, particularly in the developing countries, because it is impractical and expensive. Thus, engineering of wheat lines with high-iron and -zinc grains is a more economical and sustainable solution for micronutrient improvement and human health. As we show here, iron biofortification of wheat using gene technology is a robust method for approaching or achieving the required target levels. So far, only few genetic engineering attempts have been made to biofortify wheat for increased iron content in the grains (Drakakaki et al. 2000; Borg et al. 2012; Sui et al. 2012). We focused on two different strategies using constitutive overexpression of *NAS* and endosperm-specific expression of *FERRITIN* either alone or combined. The results we obtained for the Fer-BW lines expressing *PvFERRITIN* (Fig. 3) are similar to those previously reported (Borg et al. 2012; Sui et al. 2012). The increased zinc content in the Fer-BW lines (Fig. 3) is consistent with previous reports of expressing *FERRITIN* in both wheat and rice (Borg et al. 2012; Trijatmiko et al. 2016; Vasconcelos et al. 2003; Wirth et al. 2009).

In contrast to the Fer-BW lines, the lines overexpressing *OsNAS2* had higher iron and zinc increases in the grains, with selected lines surpassing the suggested iron target of 59 µg/g to meet the recommended 30 % EAR (Fig. 4). These lines also have higher zinc contents. Most importantly, the high whole grain iron and zinc increases are retained in the flour (Fig. 4), which usually is the part of the grain that is consumed. Overexpression of *NAS* increases the production of nicotianamine (NA) and deoxymugineic acid (DMA), ultimately facilitating the uptake and transport of PS-Fe^3+^ chelates (Wang et al. 2013; Wirth et al. 2009). *NAS* overexpression in rice also increases the expression of genes encoding the zinc transporters *OsZIP1* and *OsZIP4* (Wang et al. 2013), which can explain the increased zinc content of the transgenic lines. NA is also involved in long-distance iron transport in the phloem and translocation to the grains (Puig et al. 2007). Constitutive expression of *OsNAS2* also increased iron content in polished rice (Johnson et al. 2011; Lee et al. 2012). Moreover, mugineic acid (MA) and NA function in the uptake of other essential micronutrients, including zinc, copper, and manganese (Haydon and Cobbett 2007). Collectively, the significant increases in copper and manganese content that we find in nearly all of the Nas-BW lines in whole grains and as well as flour is consistent with the reported data (Fig. S6).

Unexpectedly, however, the expression of both *PvFERRITIN* and *OsNAS2* in wheat does not result in higher iron levels when compared to lines expressing *OsNAS2* alone. Thus, unlike rice in which the expression of *FERRITIN* and *NAS* increased iron content by sixfold in the polished rice grains (Trijatmiko et al. 2016; Wirth et al. 2009), the two genes do not seem to function synergistically in wheat. Although the reasons for this are unclear, it is plausible that iron homeostasis is controlled differently in hexaploid wheat as compared to rice and that iron accumulation is tightly regulated. Furthermore, we did not find a correlation between *PvFERRITIN* expression and iron content in both Fer-BW and FerNas-BW transgenic lines (Fig. 2) while *OsNAS2* expression in leaves correlated with the iron content of Nas-BW and FerNas-BW lines.

Together, we found that constitutive expression of *OsNAS2* alone is the preferred strategy for nutritionally relevant iron biofortification in wheat. Our data suggest that unsuccessful breeding efforts to increase iron in wheat grains could be the consequence of regulated iron uptake and transport, which can be overcome by constitutive expression of *OsNAS2.* Further detailed molecular analysis of the transgenic lines is required to understand the mechanism of metal homeostasis in wheat and how this is changed in the transgenic lines to increase grain iron content. Understanding the consequences of constitutive *OsNAS2* expression on endogenous wheat metal homeostasis-related genes will help to expand our knowledge of their interactions and will assist in improving iron in other preferred crops as well. The high iron and zinc wheat lines that we have developed are a promising breeding material after further agronomic performance testing to confirm that the iron and zinc increases observed in the greenhouse conditions are maintained in the field. The high iron and zinc traits could be easily bred into farmer-preferred and commercial cultivars, thereby reducing iron and zinc micronutrient deficiencies in affected populations.

### **Author contribution statement**

N. K. B. conceived and designed the experiments. S.S. carried out the experiments. S. S. and N. K. B. analyzed the data. S.S., N.K.B., B.K and W.G. discussed the data. S. S. and N. K. B. wrote the manuscript. W. G. and N. K. B. edited the manuscript. All authors have read the manuscript and agree with its content.

## Electronic supplementary material

Below is the link to the electronic supplementary material.

Supplementary material 1 (PDF 2019 kb)
