# Supplementary material for: Rice NICOTIANAMINE SYNTHASE 2 expression improves dietary iron and zinc levels in wheat
Source: Theor Appl Genet. 2016 Oct 8;130(2):283–92. doi: 10.1007/s00122-016-2808-x (PMC5263203; doi:10.1007/s00122-016-2808-x)
Supplement: Supplementary file 1 — Supplementary material 1 (PDF 2019 kb) [file 122_2016_2808_MOESM1_ESM.pdf]

Rice *NICOTIANAMINE SYNTHASE 2* expression improves dietary iron and zinc levels in wheat

Theoretical and Applied Genetics

Simrat Pal Singh[1], Beat Keller[2], Wilhelm Gruissem[1], and Navreet K. Bhullar[1]*
[1] Plant Biotechnology, Department of Biology, ETH Zurich, Zurich, Switzerland
[2] Institute of Plant Biology, University of Zurich, Switzerland

***Correspondence**
Dr. Navreet K. Bhullar
Plant Biotechnology,
Department of Biology
ETH Zurich
Universitaetsstrasse 2
8092 Zurich, Switzerland
bhullarn@ethz.ch

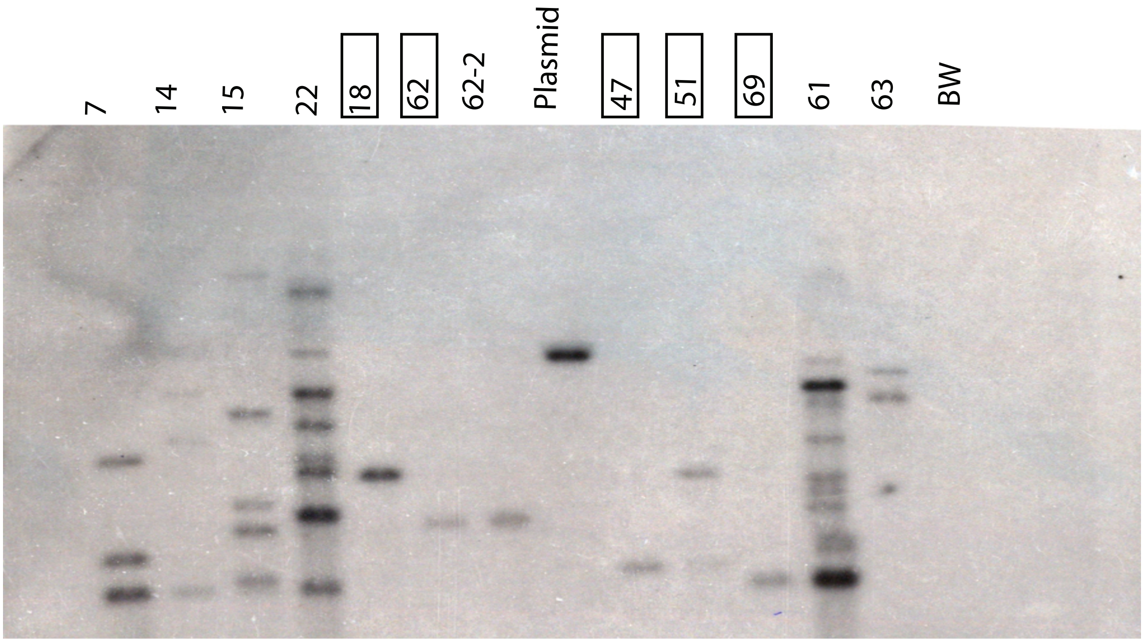

Fig. S1 Example of Southern hybridization analysis of Fer-BW transgenic lines. Genomic DNA was digested by *BglII*. Lines 18, 47, 51, 62 and 69 each contain a single copy of the transgene. Lines marked with a box were chosen for further analysis. The plasmid containing *PvFERRITIN* was used as a positive control. Bobwhite (BW) is the negative control and shows no signal for the *PvFERRITIN* specific probe

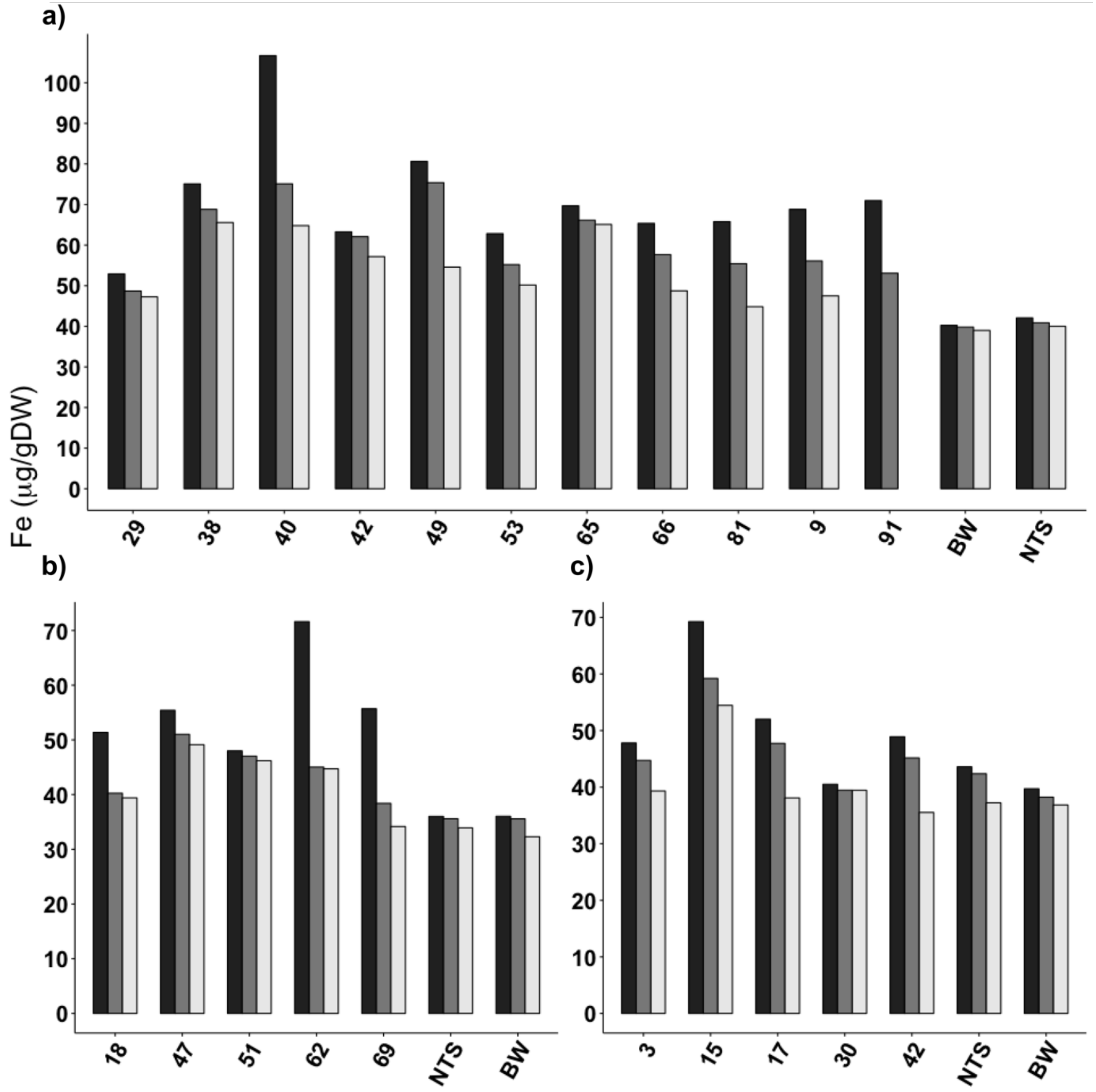

Fig. S2 Iron concentration in T3 grains of lines expressing **a)** *OsNAS2* (Nas-BW). **b)** *PvFERRITIN* (Fer-BW). **c)** *PvFERRITIN-OsNAS2* (FerNas-BW). Each bar represent individual plant for independent single insert lines. Seeds from plant represented by the black bar were used to grow the next generation (T3) to harvest T4 grains (data presented in the manuscript). BW(Bobwhite); NTS (non-transgenic sibling)

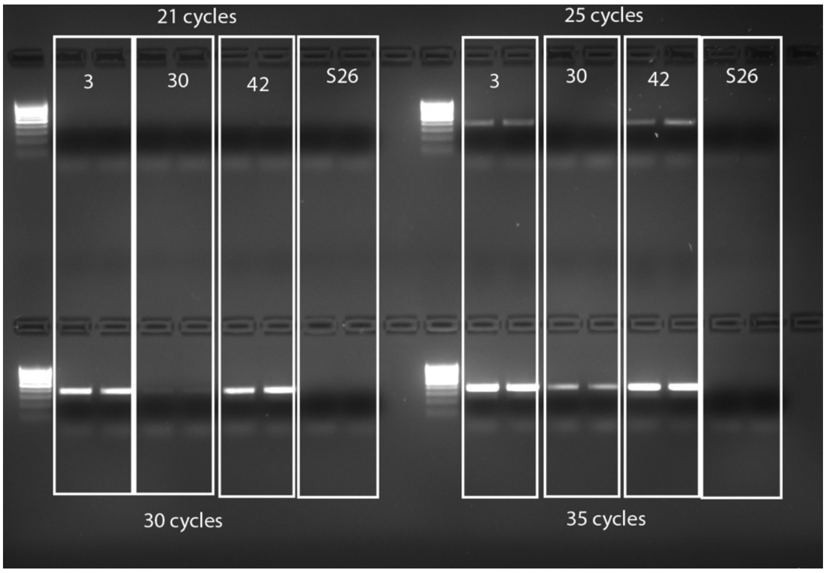

Fig. S3 Semi-quantitative RT PCR for detection of *FERRITIN* expression. Semi-quantitative RT PCR was done on samples 3, 30 and 42 among the FerNas-BW lines using primers listed in Table S1. The results are captured after 21, 25, 30 and 35 cycles

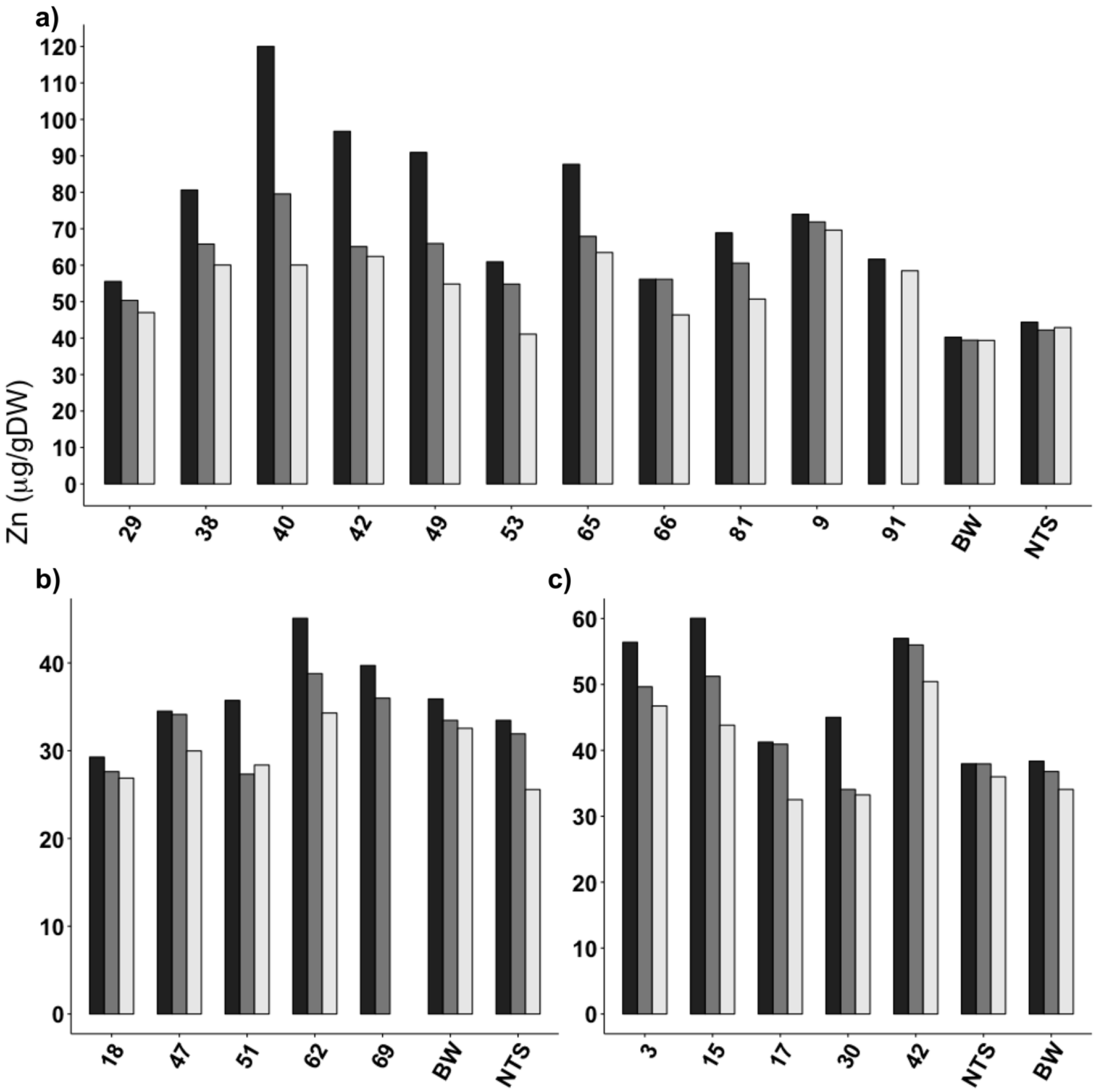

Fig. S4 Zinc concentration in T3 grains of lines expressing **a)** *OsNAS2* (Nas-BW). **b)** *PvFERRITIN* (Fer-BW). **c)** *PvFERRITIN-OsNAS2* (FerNas-BW). Each bar represent individual plant for independent single insert lines. BW(Bobwhite); NTS (non-transgenic sibling)

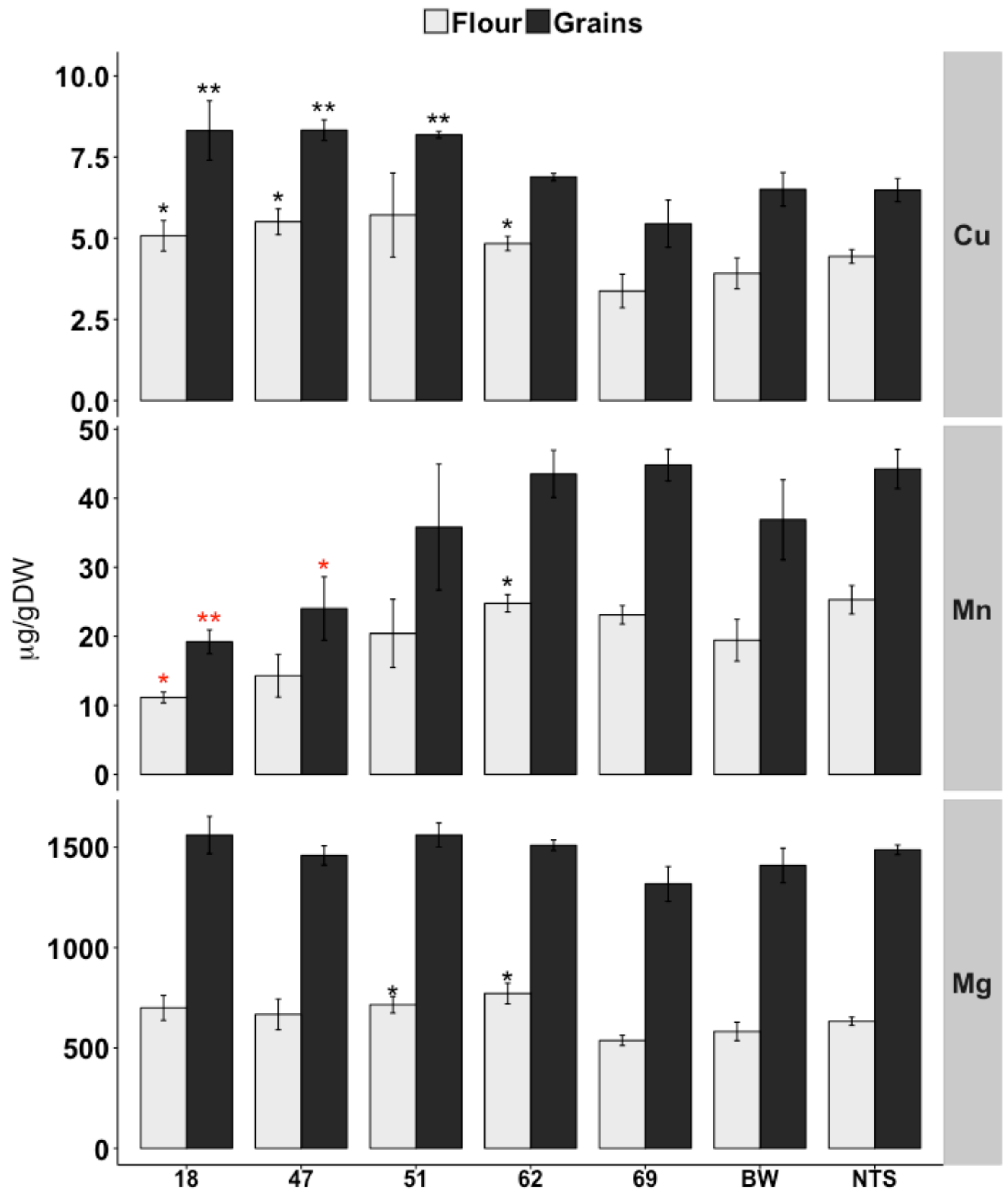

Fig. S5 Metal content in T4 grains and flour of lines expressing *PvFERRITIN* (Fer-BW). Values are the mean of three biological replicates (±SD). Black and red asterisks above the bars indicate statistically higher and lower significant values calculated using Student's T test, respectively, in comparison to the control line Bobwhite (BW) (*P < 0.05; **P < 0.01). NTS, non-transgenic sibling. Cu, copper, Mn, manganese, Mg, magnesium

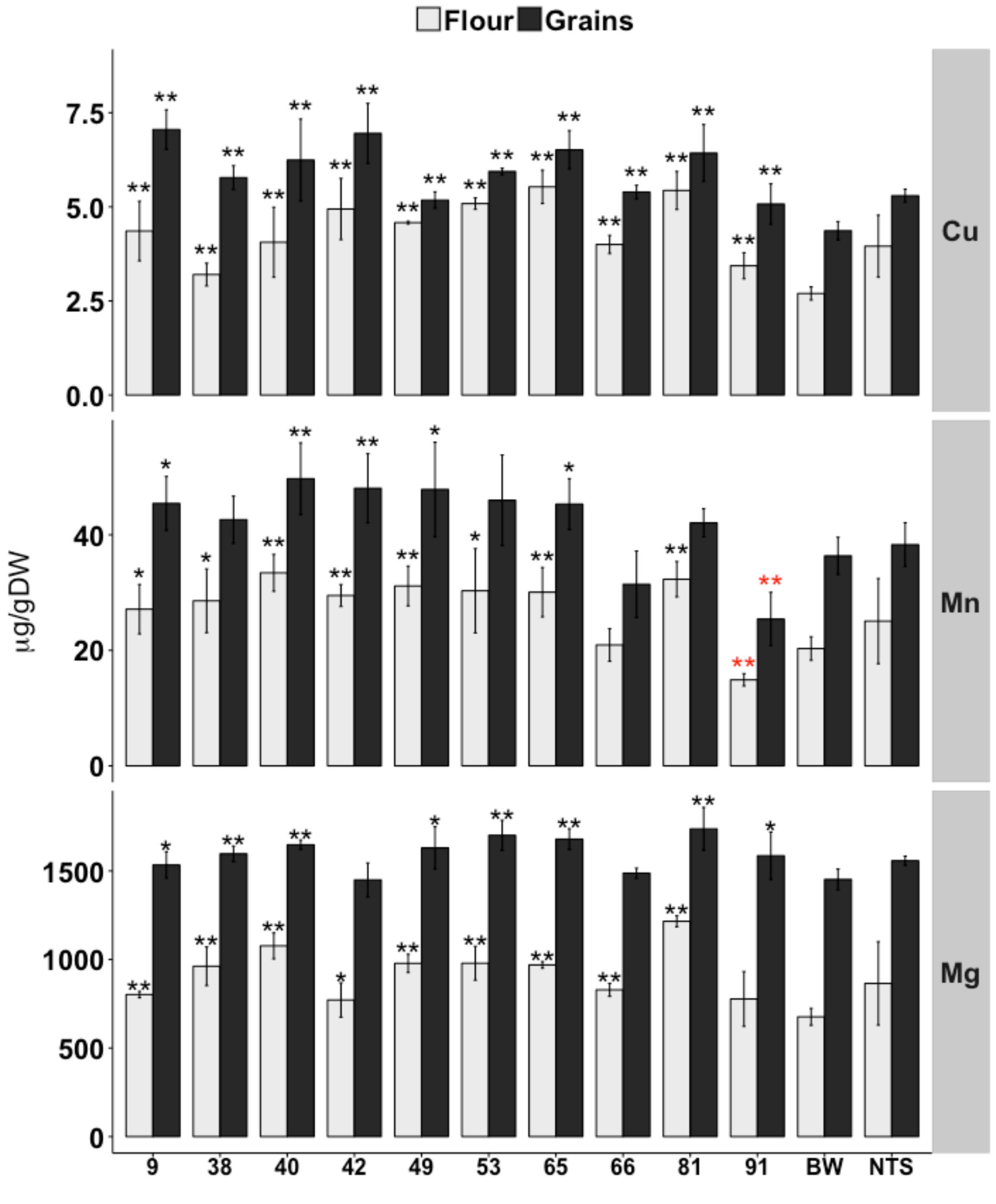

Fig. S6 Metal content in T4 grains and flour of lines expressing *OsNAS2* (Nas-BW). Values are the mean of three biological replicates (±SD). Black and red asterisks above the bars indicate statistically higher and lower significant values calculated using Student's T test, respectively, in comparison to the control line Bobwhite (BW) (*P < 0.05; **P < 0.01). NTS, non-transgenic sibling

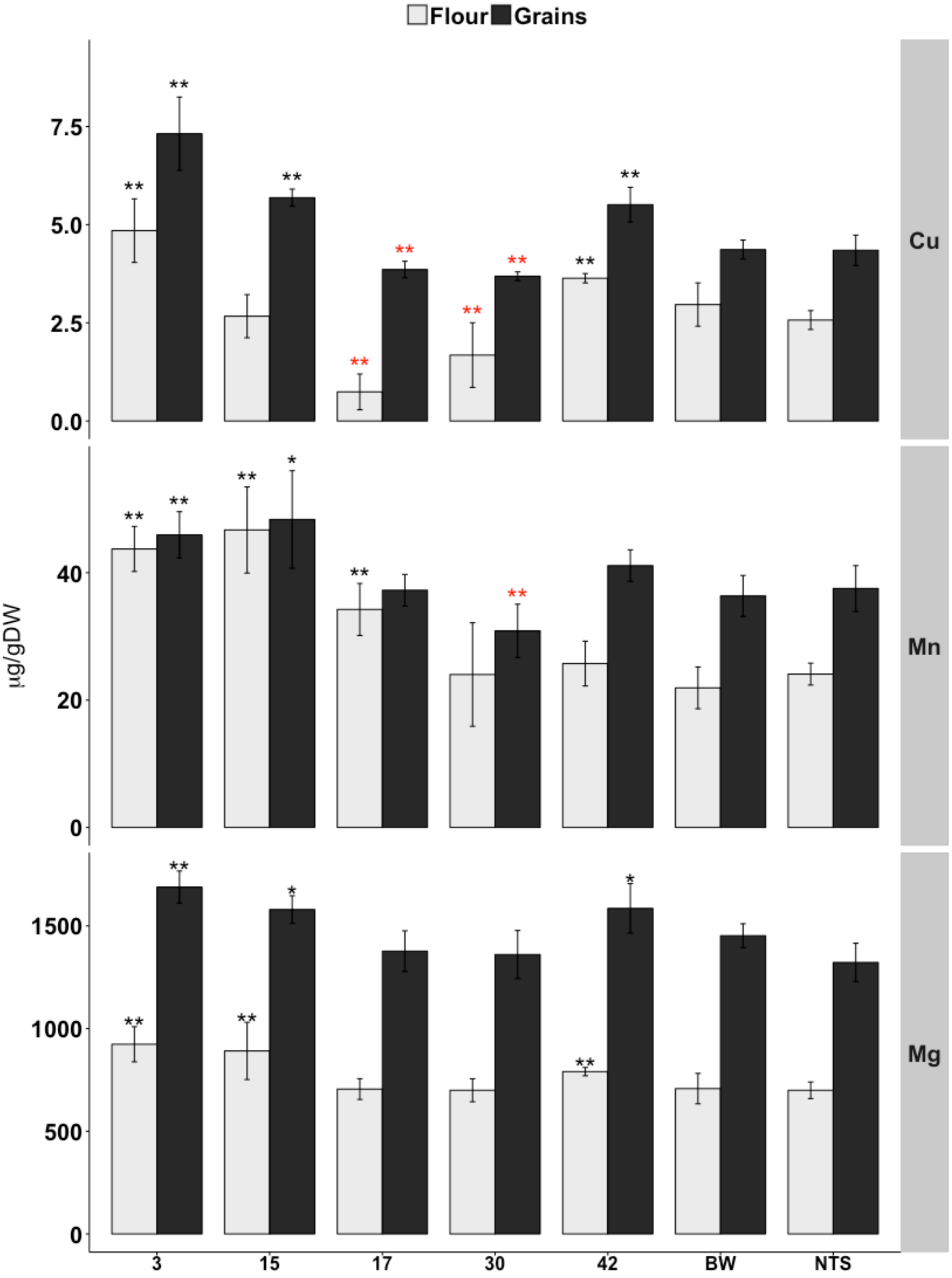

Fig. S7 Metal content in T4 grains and flour of lines expressing *PvFERRITIN-OsNAS2* (FerNas-BW). Values are the mean of three biological replicates (±SD). Black and red asterisks above the bars indicate statistically higher and lower significant values calculated using Student's T test, respectively, in comparison to the control line Bobwhite (BW)(*P < 0.05; **P < 0.01). NTS, non-transgenic sibling

Table S1 Primers used for PCR screening, generation of probe for Southern hybridization, and for quantitative gene expression analysis (qRT-PCR)

Gene	Forward primer	Reverse primer
**PCR screening and Southern hybridisation probe**		
PvFERRITIN	CTCTTTTTCCAACCGATCCA	AAGCTTTTCAGCGTGCTCTC
OsNAS2	CAAGTGCTGCAAGATGGA	GCGGGACTCTAATCATAAAA
**qRT-PCR**		
PvFERRITIN	AAGCAGGAACCTTGGTGT	AGGGTACATTCTTGATCG
OsNAS2	GCAAGTGCTGCAAGATGG	CCGGATCAGACGGATAGC
Ta.22845	AAGAAGCAAGAGCTCGGATTT	TGCACCGTTGAAATCATCA

Table S2 Phenotypic performance of greenhouse-grown T3 generation Fer-BW transgenic lines

Plant line	Days to flowering	Height (cm)	1000 GW (g)	Tiller nr.
18	96 ± 3**	71.8 ± 7.9	33.8 ± 0.8	10 ± 1**
47	91.7 ± 4.6	55.9 ± 6	34.6 ± 1.9	7.7 ± 0.6**
51	89 ± 7.5	65.4 ± 2.1	34.6 ± 3.3	12.7 ± 1.5
62	87.7 ± 7.4	58.8 ± 4.1	37 ± 1.3	10 ± 2.6**
69	99.3 ± 3.5**	70 ± 3.2	37.5 ± 0.3	10.3 ± 0.6**
NTS	92 ± 4.6	71 ± 4.7	36.2 ± 0.6	15.7 ± 1.2
BW	83 ± 3.5	64.8 ± 5.2	36.4 ± 1.6	15 ± 1

Values are the average of three biological replicates (± standard deviation). Transgenic plants were compared to Bobwhite (BW). Black and red asterisks indicate statistically higher and lower significant values calculated using Student's T test, respectively (*P < 0.05; **P < 0.01)

Table S3 Phenotypic performance of greenhouse-grown T3 generation FerNas-BW transgenic lines

Plant line	Days to flowering	Height (cm)	1000 GW (g)	Tiller nr.
3	107±3.5	51.3±2	29.2±2.4	20.3±9.9
15	94±4	48.6±1.5	29.7±2.8	20±5.6
17	102±5.6	53.9±3.3	30.5±0.4	16.7±3.2
30	103.7±3.8	54.5±8.1	26.2±6.7	13.3±3.1
42	100.3±9.3	56.9±1,*	33.4±1.8	19.7±2.3
NTS	100±2.6	46.7±5.3	32.5±1	20±3.6
BW	101.7±0.5	49.6±1.9	29.2±1.9	16±3

Values are the average of three biological replicates (± standard deviation). Transgenic plants were compared to Bobwhite (BW). Black asterisks indicate statistically higher significant values calculated using Student's T test, (*P < 0.05; **P < 0.01)
